# The Alternative Splice Variant of Protein Tyrosine Kinase 6 Negatively Regulates Growth and Enhances PTK6-Mediated Inhibition of β-Catenin

**DOI:** 10.1371/journal.pone.0014789

**Published:** 2011-03-30

**Authors:** Patrick M. Brauer, Yu Zheng, Mark D. Evans, Carmen Dominguez-Brauer, Donna M. Peehl, Angela L. Tyner

**Affiliations:** 1 Department of Biochemistry and Molecular Genetics, University of Illinois College of Medicine, Chicago, Illinois, United States of America; 2 Department of Urology, Stanford University School of Medicine, Stanford, California, United States of America; Kimmel Cancer Center, United States of America

## Abstract

Protein tyrosine kinase 6 (PTK6), also called breast tumor kinase (BRK), is expressed in epithelial cells of various tissues including the prostate. Previously it was shown that PTK6 is localized to epithelial cell nuclei in normal prostate, but becomes cytoplasmic in human prostate tumors. PTK6 is also primarily cytoplasmic in the PC3 prostate adenocarcinoma cell line. Sequencing revealed expression of wild type full-length PTK6 transcripts in addition to an alternative transcript lacking exon 2 in PC3 cells. The alternative transcript encodes a 134 amino acid protein, referred to here as ALT-PTK6, which shares the first 77 amino acid residues including the SH3 domain with full length PTK6. RT-PCR was used to show that *ALT-PTK6* is coexpressed with full length *PTK6* in established human prostate and colon cell lines, as well as in primary cell lines derived from human prostate tissue and tumors. Although interaction between full-length PTK6 and ALT-PTK6 was not detected, ALT-PTK6 associates with the known PTK6 substrates Sam68 and β-catenin in GST pull-down assays. Coexpression of PTK6 and ALT-PTK6 led to suppression of PTK6 activity and reduced association of PTK6 with tyrosine phosphorylated proteins. While ALT-PTK6 alone did not influence β-catenin/TCF transcriptional activity in a luciferase reporter assay, it enhanced PTK6-mediated inhibition of β-catenin/TCF transcription by promoting PTK6 nuclear functions. Ectopic expression of ALT-PTK6 led to reduced expression of the β-catenin/TCF targets Cyclin D1 and c-Myc in PC3 cells. Expression of tetracycline-inducible ALT-PTK6 blocked the proliferation and colony formation of PC3 cells. Our findings suggest that ALT-PTK6 is able to negatively regulate growth and modulate PTK6 activity, protein-protein associations and/or subcellular localization. Fully understanding functions of ALT-PTK6 and its impact on PTK6 signaling will be critical for development of therapeutic strategies that target PTK6 in cancer.

## Introduction

Protein tyrosine kinase 6 (PTK6), also known as breast tumor kinase (BRK) in humans and Src-related intestinal kinase (Sik) in the mouse, is structurally related to Src, but is a member of a distinct family [1], [2]. PTK6 was first discovered in a screen for tyrosine kinases expressed in human cultured melanocytes [3], and it was later cloned from human breast cancer cells [4] and the gastrointestinal tract of the mouse [5]. PTK6 expression has been detected in differentiated epithelial cells of the gastrointestinal tract [5], [6], [7], [8], [9], oral epithelium [10], prostate [8], [11], skin [3], [6], [12], and lymphocytes [13]. Studies suggest that PTK6 promotes differentiation in normal epithelia [12], [14], [15]. In normal intestine, PTK6 also negatively regulates growth [15] and promotes DNA-damage induced apoptosis [16], [17].

Although PTK6 is not expressed in normal mammary gland or ovarian tissue [7], [18], it is expressed in a high percentage of breast [4], [18], [19], [20] and ovarian cancers that have been examined [21]. Several studies indicate that PTK6 promotes oncogenic signaling in breast cancer cells (reviewed in [1], [22]). While PTK6 is expressed in normal prostate, the intracellular localization of PTK6 changes in prostate cancers; it is nuclear in normal prostate epithelial cells, but relocalizes to the cytoplasm in prostate cancer [11]. A variety of data now suggest that functions of PTK6 may depend on its intracellular localization, access to specific substrates and the tissue in which it is expressed ([23], [24], [25] and reviewed in [1]).

The Wnt/β-catenin/TCF signaling pathway plays an important role in the growth of many different cancers including those of the prostate (reviewed in [26], [27]). We recently demonstrated that β-catenin is a direct substrate of PTK6, and that PTK6 regulates β-catenin transcriptional activity in the human SW620 colon cancer cell line, and in the mouse intestine [23]. The precise functions that β-catenin signaling plays in prostate cancer are not well understood, and its complexity is further compounded by crosstalk of β-catenin with multiple signaling pathways involving factors such as the androgen receptor [28], IGF-1 (insulin-like growth factor 1) [29], [30], AKT [29], [31], osteopontin [32], [33], and FoxO (Forkhead box O) [34], [35], [36]. PTK6 may also be activated by IGF-1 [37], [38] and osteopontin [39]. In addition, PTK6 regulates AKT [15], [17], [40], [41], FKHR/FoxO1 [15], [41] and FoxO3 [42]. There can also be competition between different signaling pathways for β-catenin interactions, such as FoxO and TCF, resulting in transactivation of different genes ([43] and reviewed in [35], [44]). Downregulation of Wnt/β-catenin signaling in cancer is an attractive therapeutic target, and may be achieved by treatment with various inhibitors (reviewed in [45]).

An alternatively spliced PTK6 transcript that encodes a 15 kDa protein including the PTK6 SH3 domain and a unique proline-rich carboxy-terminus was previously detected in the T47D human breast cancer cell line [46]. Functions of this alternative PTK6 isoform, originally called λm5 but referred to here as ALT-PTK6 (alternative PTK6 isoform), have not been explored. We found that ALT-PTK6 transcripts are present in human prostate epithelial cell lines derived from normal prostate and prostate adenocarcinomas, as well as in a variety of human cell lines. We have examined potential roles of ALT-PTK6 and found that PTK6 functions, including its ability to regulate β-catenin/TCF transcription, can be influenced by ALT-PTK6. Knowing the functions of ALT-PTK6 may be important for devising PTK6 targeted therapies.

## Results

### Two PTK6 transcripts are expressed in prostate and colon tumor cell lines

**Figure 1:**
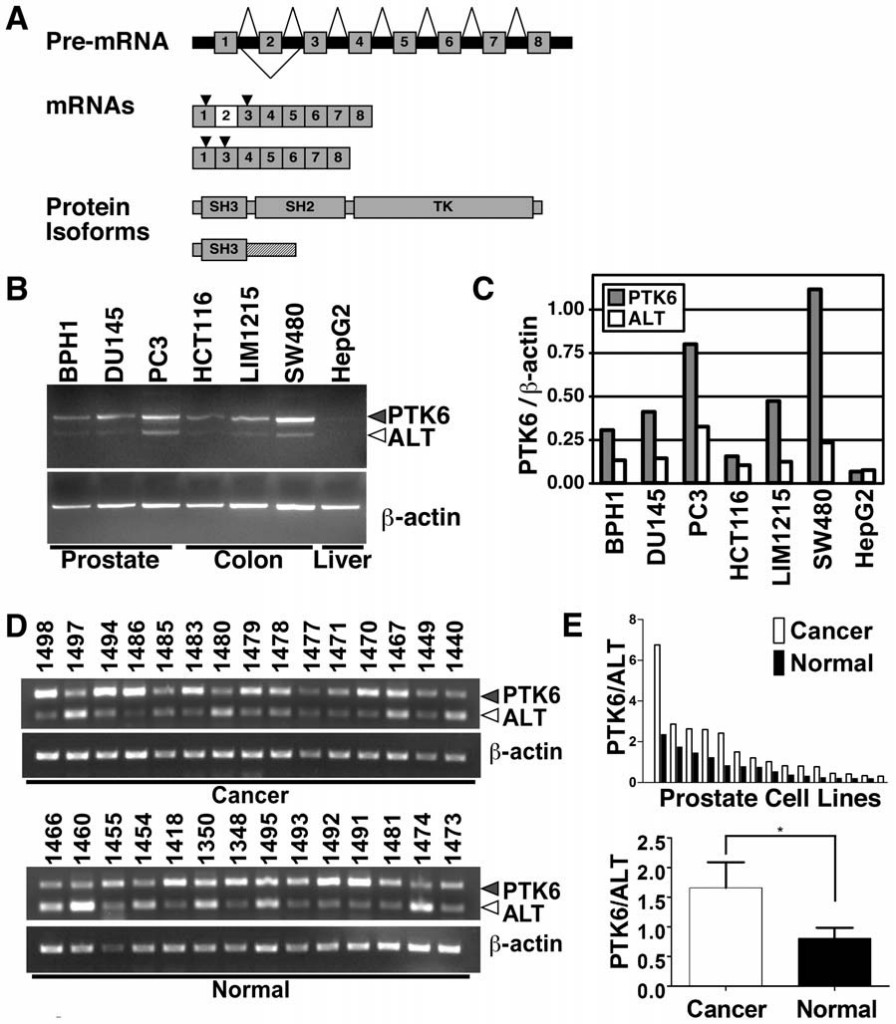
Expression of *PTK6* transcripts. A) *PTK6* gene structure and its products. The *PTK6* gene contains 8 exons. The transcript encoding full length PTK6 contains all eight exons, but alternative splicing results in exclusion of exon 2 in *ALT-PTK6*. The *PTK6* transcript encodes the full, catalytically active 451 amino acid protein, while the *ALT-PTK6* transcript encodes a protein that shares the first 77 amino acids (including the amino-terminus and SH3 domain) with full length protein, and also contains a 57 amino acid novel, truncated proline rich carboxyl-terminus due to a frame shift (striped area). Primers for semi-quantitative PCR that distinguish between full length and *ALT-PTK6* are specific to exons 1 and 3 (arrows). B) Semi-quantitative RT-PCR analysis shows that both *PTK6* and *ALT-PTK6* (ALT) transcripts are expressed in human prostate and colon cell lines. The HepG2 liver cancer cell line does not express *PTK6* and was used as a negative control. C) Quantification of *PTK6* (grey bars) and *ALT-PTK6* (white bars) shown in B normalized to a β-actin loading control shows higher levels of *PTK6* expression compared with *ALT-PTK6* transcript. D) Both full length and ALT-PTK6 transcripts are expressed in primary cultures of normal human prostate epithelial cells from the peripheral zone (Normal), as well as in primary epithelial cultures from prostate adenocarcinomas (Cancer). E) Quantification of *PTK6* and *ALT-PTK6* transcripts expressed in normal prostate epithelial cells (black bars) and prostate tumor cells (white bars) shown in D. The ratio of PTK6 over ALT-PTK6 from individual samples was graphed in order from highest to lowest within cancer and normal sample groups (Top). The cancer and normal sample group mean was calculated with standard error of mean (SEM) as error bars (bottom). Prostate tumor cells have a higher *PTK6* to *ALT-PTK6* ratio than normal prostate cells. The asterisk (*) corresponds to a P-value of <0.05.

Altered intracellular localization of PTK6 in prostate cancer cells, including the PC3 cell line [11], raised the possibility that the *PTK6* gene might harbor mutations that contribute to altered PTK6 localization. We analyzed the sequence of PCR-amplified reverse-transcribed *PTK6* mRNA and compared it to the *PTK6* cDNA sequence in the database (accession NM_005975). No mutations within the *PTK6* sequences of PC3 cells were identified, however the expression of the *ALT-PTK6* transcript was confirmed in PC3 cells (Figure 1). The RNA and protein expression of both full-length PTK6 and ALT-PTK6 was previously reported in the T47D human breast cancer cell line [46].

To examine expression of the *ALT-PTK6* transcript in a variety of human cell lines, primers specific to exon 1 (forward) and 3 (reverse) were designed to distinguish the PCR products from full length *PTK6* (407 bp) and *ALT-PTK6* (285 bp) transcripts. Both *PTK6* and *ALT-PTK6* transcripts were expressed concomitantly in human prostate (BPH1, DU145, PC3) and colon (HCT116, LIM1215, SW480) cell lines (Figure 1B). Expression of β-actin served as a control. Levels of *PTK6* transcripts containing exon 2, which includes the full length *PTK6* transcript but not *ALT-PTK6*, and exon 8, which would encompass both full-length *PTK6* and *ALT-PTK6,* were also quantified using real time qRT-PCR and primers specific to either exon 2 or exon 8 (Figure S1 A). Values for each cell line were normalized to cyclophilin; to account for differences in primer efficiencies, transcript levels determined by primers specific to exons 2 and 8 were normalized to each other using the pcDNA3-PTK6 expression construct, which contains both exons 2 and 8 at a 1∶1 ratio (Figure S1 B). The ratios of exon 8 to exon 2 were also determined (Figure S1 C). The HepG2 liver cancer cell line does not express *PTK6* and is a negative control.

Expression of *PTK6* and *ALT-PTK6* was also analyzed in primary cultures of normal epithelial cells derived from fourteen prostates (peripheral zone) and epithelial cells from fifteen prostate adenocarcinomas using PCR (Figure 1D). Both full-length and ALT-PTK6 transcripts were observed in cell lines derived from normal prostate and prostate tumors. However, prostate cancer cells express higher levels of the transcript encoding full length PTK6 (Figure 1D and E). We have found that the protein encoded by the full length transcript promotes growth of PC3 cells when it is localized to the cytoplasm [25]. Unfortunately, neither commercial antibodies specific for the amino-terminus shared by PTK6 and ALT-PTK6 that were available at the time of this study, nor the antibody stocks that originally detected expression of both proteins [46] (a kind gift from Dr. Mark Crompton) detected either endogenous protein in our hands. Despite multiple attempts, we were unsuccessful in raising monoclonal antibodies against ALT-PTK6.

### ALT-PTK6 inhibits phosphorylation of PTK6

**Figure 2:**
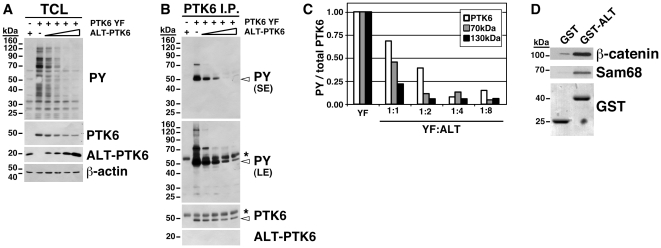
ALT-PTK6 inhibits PTK6 phosphorylation and PTK6 association with other tyrosine-phosphorylated proteins. A) HEK293 cells were co-transfected with constitutively active PTK6 YF and ALT-PTK6. A dose-dependent reduction in tyrosine phosphorylation of proteins was detected in total cell lysates (TCL). Empty vector was used to keep the total levels of transfected plasmid DNA equal. B) Immunoprecipitation of comparable levels of PTK6 shows reduced association between PTK6 and tyrosine-phosphorylated proteins (PY), in addition to reduced tyrosine phosphorylation of PTK6 itself. Arrows correspond to PTK6, and asterisks (*) denote IgG from the immunoprecipitation. Short (SE) and long (LE) exposures are shown. C) Quantification of tyrosine-phosphorylated PTK6 and several binding partners normalized to total pulled-down PTK6 shows that ALT-PTK6 reduces PTK6 interactions by greater than 50% even at low ratios. D) GST pull-down experiments were performed with GST alone or GST-ALT-PTK6. Association of known PTK6 substrates β-catenin [23] and SAM68 [50] with ALT-PTK6 was detected by immunoblotting.

Although ALT-PTK6 was first described in 1997 [46], the potential significance of its expression has not been studied. To better understand the functions of ALT-PTK6, we generated a Myc-epitope tagged ALT-PTK6 expression construct. HEK293 cells that do not express endogenous PTK6 were transiently transfected with a constant amount of constitutively active PTK6 YF and increasing amounts of ALT-PTK6. Immunoblotting showed decreasing tyrosine phosphorylation of proteins in total cell lysates with increasing ALT-PTK6 (Figure 2A). Although we detected some decrease in the levels of ectopic PTK6 YF when increasing levels of ALT-PTK6 were introduced, greater inhibition of overall tyrosine phosphorylation was observed. Similar results were observed following co-transfection of ALT-PTK6 with PTK6 YF in the PC3 cell line (data not shown). Immunoprecipitation of equivalent levels of PTK6 YF revealed that addition of ALT-PTK6 resulted in decreased association of PTK6 with tyrosine-phosphorylated proteins (Figure 2B). Even at a PTK6 YF:ALT-PTK6 ratio of 1∶1, association of PTK6 with phosphotyrosine proteins decreased. Tyrosine phosphorylation of a band near 50 kDa that corresponds to PTK6 also decreased, and higher levels of ALT-PTK6 reduced phospho-PTK6 levels even further. The impact that ALT-PTK6 expression had on protein tyrosine phosphorylation was quantified in Figure 2C. As is the case with Src, PTK6 activity is regulated by intramolecular interactions of the SH2 domain and phosphorylated carboxy-terminal tyrosine, as well as by SH3 domain binding to the proline rich linker region between the SH2 and catalytic domains [47], [48], [49]. However, we did not detect ALT-PTK6 association with full length PTK6 in co-immunoprecipitation experiments (Figure 2B).

While the major tyrosine phosphorylated proteins associated with PTK6 were not identified in Figure 2B, we did detect ALT-PTK6 association with known PTK6 substrates and interacting proteins. Total cell lysates prepared from PC3 cells were incubated with GST or GST-tagged ALT-PTK6 fusion protein and GST pull down assays were performed. Association of the PTK6 substrates Sam68 [50] and β-catenin [23] with ALT-PTK6 was readily detected (Figure 2D). These data demonstrate that ALT-PTK6 participates in protein-protein interactions and may compete with the full-length protein.

### ALT-PTK6 enhances nuclear functions of PTK6

**Figure 3:**
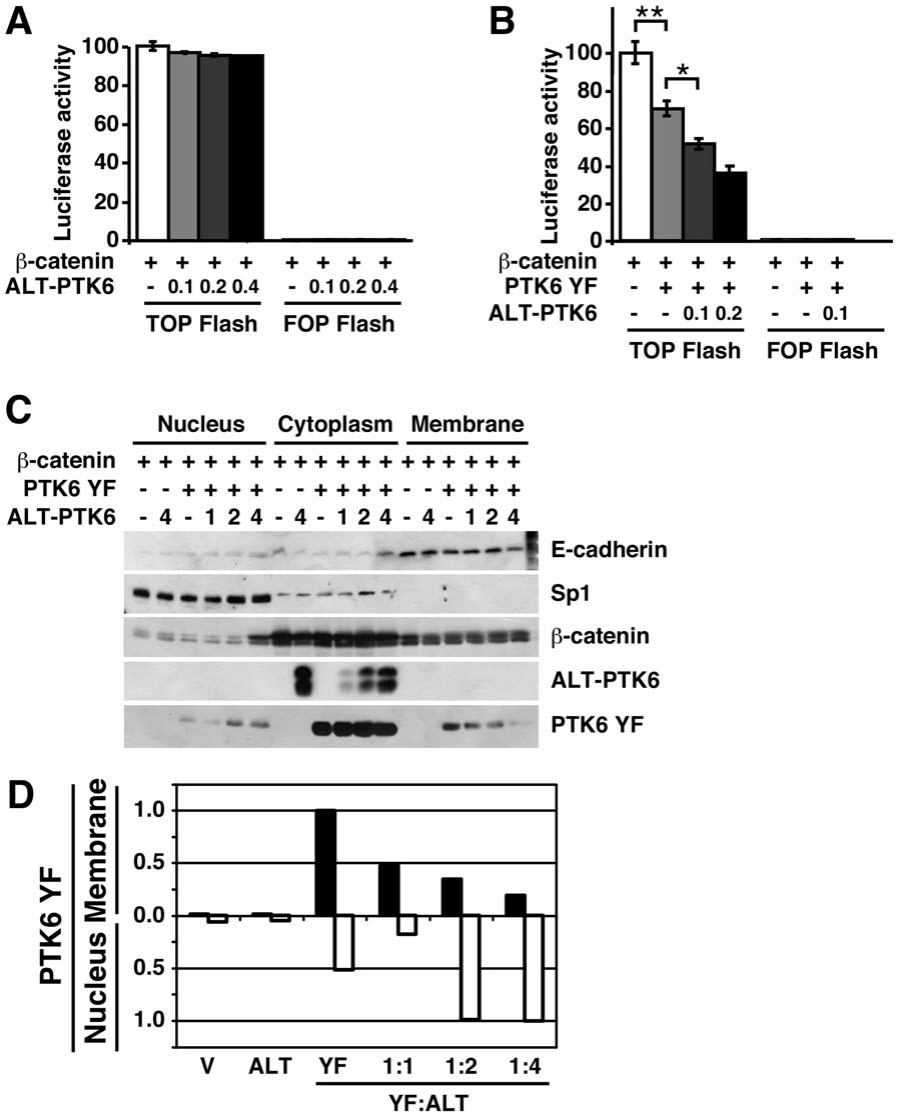
ALT-PTK6 enhances PTK6-mediated inhibition of β-catenin/TCF transcriptional activity by promoting the nuclear function of PTK6. A) HEK293 cells were transfected with the Super 8x TOPFlash (TOPFlash) luciferase reporter construct, the control luciferase reporter (FOPFlash), β-catenin, vector (-), or ALT-PTK6. β-catenin/TCF transcriptional activity in HEK293 cells was not affected when ALT-PTK6 was expressed in the absence of PTK6. B) Transfections were performed as in (A), with the addition of PTK6 YF as indicated. ALT-PTK6 promotes the PTK6 YF mediated inhibition of β-catenin transcriptional activity. Asterisks correspond to P-values of <0.005 (**), and <0.01 (*). C) Immunoblot analysis of fractionated HEK293 cells expressing PTK6 YF and ALT-PTK6. ALT-PTK6 enhances nuclear localization of PTK6. β-catenin transcriptional activity is inhibited by PTK6 in the nucleus, but membrane-associated PTK6 results in increased β-catenin transcriptional activity [23]. E-cadherin and Sp1 were used as loading controls. D) Quantification of membrane and nuclear pools of PTK6 YF. Increasing levels of ALT-PTK6 results in reduced PTK6 in the membrane fraction (black bars) and increased nuclear PTK6 (white bars). Membrane PTK6 was normalized to E-cadherin, and nuclear PTK6 was normalized to Sp1.

Recently, full length PTK6 was shown to inhibit β-catenin/TCF transcription [23]. Although transcriptional inhibition of β-catenin did not require PTK6 kinase activity, we observed direct interaction between PTK6 and β-catenin [23]. To determine if ALT-PTK6 can influence β-catenin/TCF mediated transcription we used the Super 8X TOPFlash (TOPFlash) luciferase reporter construct [51]. HEK293 cells were co-transfected with TOPFlash, β-catenin and increasing levels of ALT-PTK6 plasmids. Addition of ALT-PTK6 did not alter TOPFlash reporter activity (Figure 3A). As expected, co-transfection of β-catenin and constitutively active PTK6 YF repressed transcription (Figure 3B). Interestingly, when increasing levels of ALT-PTK6 were co-expressed with TOPFlash, β-catenin, and PTK6 YF, luciferase reporter activity was further repressed (Figure 3B).

In our previous work, we showed that the subcellular localization of PTK6 influenced the ability of PTK6 to regulate β-catenin transcriptional activity [23]. To determine if ectopic ALT-PTK6 expression had an impact on the intracellular localization of PTK6 in HEK293 cells, transfected cells were fractionated and localization of PTK6 and ALT-PTK6 was examined by immunoblotting. PTK6 YF and ALT-PTK6 were detected using Myc-epitope specific antibody, and the majority of both PTK6 isoforms localized to the cytoplasm (Figure 3C). However, increased expression of ALT-PTK6 resulted in a dose-dependent decrease of PTK6 YF at the membrane and, more importantly, an increase in nuclear PTK6 YF (Figure 3C and D).

### ALT-PTK6 enhances PTK6-mediated repression of β-catenin/TCF target genes in prostate tumor cells

**Figure 4:**
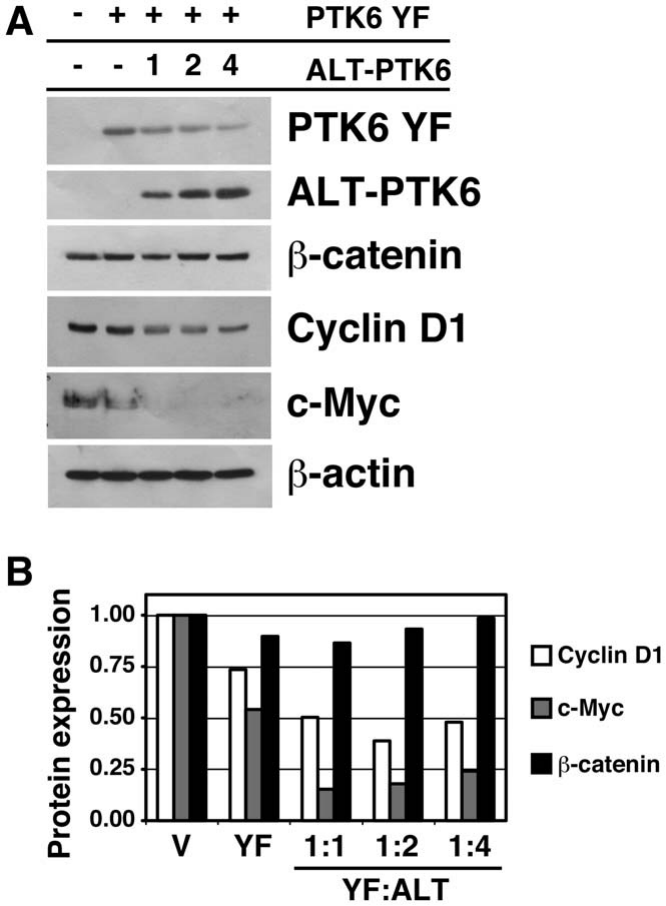
ALT-PTK6 decreases expression of β-catenin/TCF transcriptional targets in prostate tumor cells. A) Co-transfection of PTK6 YF and ALT-PTK6 expression constructs into PC3 cells results in reduced expression of the β-catenin/TCF transcriptional targets Cyclin D1 and c-Myc. Levels of endogenous β-catenin were not affected, and β-actin was used as a loading control. The pcDNA3 vector was used to keep the total amount of transfected DNA equal. B) Quantification of Cyclin D1 (white bars), c-Myc (grey bars), and β-catenin (black bars) normalized to β-actin.

Enhanced nuclear localization of PTK6 can contribute to the repression of β-catenin/TCF targets [23]. Since ALT-PTK6 led to repression of the β-catenin/TCF luciferase reporter (Figure 3B), we examined the impact of ALT-PTK6 expression on the growth promoting targets of β-catenin/TCF. To determine if PTK6 and ALT-PTK6 can regulate β-catenin/TCF signaling in prostate cancer cells, PC3 cells were transfected with a constant amount of PTK6 YF and increasing ALT-PTK6 (Figure 4A). Repression of the β-catenin/TCF transcriptional targets Cyclin D1 and c-Myc were observed in cells transfected with PTK6 YF (Figure 4A, - ALT-PTK6), similar to what we previously observed in the SW620 colon cancer cell line [23]. Introduction of ALT-PTK6 resulted in enhanced repression of the β-catenin target genes compared with PTK6 YF alone. Levels of β-catenin were not affected. Levels of Cyclin D1, c-Myc and β-catenin were quantified and normalized to β-actin (Figure 4B).

### ALT-PTK6 inhibits proliferation and colony formation of PC3 prostate tumor cells

**Figure 5:**
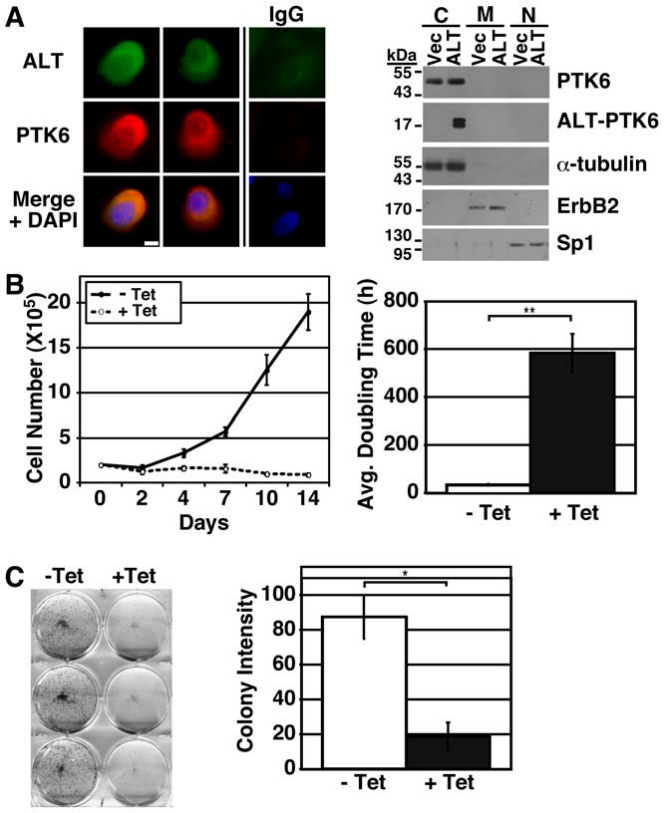
ALT-PTK6 inhibits the growth of PC3 prostate tumor cells. A) The intracellular localization of tetracycline-induced ALT-PTK6 was examined by immunofluorescent staining (left) and fractionation (right) using Myc-epitope specific antibody. Immunofluorescent staining shows ALT-PTK6 (green) is cytoplasmic in PC3 cells that were tetracycline-induced for ALT-PTK6 expression. The IgG control used normal serum in place of Myc-epitope specific antibody to show background staining in tetracycline-induced cells. Nuclei are visualized with DAPI (blue). The size bar corresponds to 20 µm. Fractionation of PC3 cells following tetracycline induction of ALT-PTK6 (ALT) shows that ALT-PTK6 localizes to the cytoplasmic compartment. Tetracycline-treated PC3 cells containing vector were used as a control (Vec). Tubulin, ErbB2, and Sp1 serve as markers for the cytoplasmic, membrane, and nuclear fractions respectively. B) A significant decrease in PC3 cell growth is detected when ALT-PTK6 is induced (dotted line, + Tet) compared with uninduced (solid line, - Tet) conditions. C) Colony formation assay of stable ALT-PTK6 inducible PC3 cells which generate colonies when untreated (-Tet). Colony formation is inhibited when ALT-PTK6 expression was induced (+Tet). Asterisks correspond to P-values of <0.01 (*) and <0.007 (**).

Activation of β-catenin/TCF regulated transcription often contributes to tumor cell growth [52]. To determine if ALT-PTK6 can affect growth, stable PC3 cell lines expressing tetracycline-inducible ALT-PTK6 were generated. Indirect immunofluorescence was performed to examine localization of induced ALT-PTK6 (ALT) and endogenous full length PTK6, and we found that both proteins are predominantly cytoplasmic (Figure 5A, left panel). Expression levels and cytoplasmic localization of ALT-PTK6 were also examined by cell fractionation followed by immunoblotting (Figure 5A, right panel). Induced expression of ALT-PTK6 did not have a detectable impact on endogenous PTK6 expression or intracellular localization in PC3 cells (Figure 5A). However, PC3 cells expressing ALT-PTK6 had reduced proliferation compared with the uninduced control (Figure 5B). In agreement with the reduction in cell proliferation data, colony formation of ALT-PTK6 expressing cells was also significantly inhibited compared with uninduced control cells (Figure 5C). These results suggest that ALT-PTK6 has a negative effect on growth in prostate cancer cells, which correlates with its ability to enhance repression of β-catenin/TCF regulated transcription (Figures 3 and 4).

## Discussion

Our work, as well as the work of others, suggests that PTK6 subcellular localization influences its functions [11], [23], [24], [25], [53]. PTK6 is localized to the cytoplasm of PC3 cells and this cell line provides a model for assessing the mechanisms underlying altered PTK6 intracellular localization in prostate cancer. While a mutation within the *PTK6* gene was not identified, we found that PC3 cells express both wild type full length *PTK6* and alternatively spliced *ALT-PTK6* transcripts. The exclusion of exon 2 results in a frame shift producing a transcript with an early stop codon, which encodes a 134 amino acid protein that shares its amino terminus and SH3 domain with full length PTK6, and contains a novel proline rich carboxy-terminal sequence (Figure 1A) [46]. Analysis of *PTK6* transcripts expressed in BPH1, DU145 and PC3 prostate cell lines, and HCT116, LIM1215 and SW480 colon cancer cell lines showed the presence of full-length and alternatively spliced transcripts (Figure 1B and Figure S1 B). *ALT-PTK6* expression does not appear to be restricted by cell type, and is expressed in breast [46], prostate, and colon tumor cell lines (Figure 1B). Both transcripts were detected in normal prostate cells, as well as prostate tumor cells, suggesting that *ALT-PTK6* is not restricted to cancer cells (Figure 1C). Prostate tumor cells had a higher *PTK6* to *ALT-PTK6* ratio than normal prostate cells. If protein levels are representative, this suggests an additional mode of aberrant PTK6 function in prostate cancer, along with upregulation and mislocalization. Interestingly, PTK6 itself could have an impact on the regulation of alternative splicing, since PTK6 substrates include a number of RNA binding proteins such as Sam68, SLM1, and SLM2 [50], [53], as well as PSF [54] that regulate splicing [55], [56], [57], [58].

A variety of proteins contain catalytic domains, which are essential for specific enzymatic functions. Other regions mediate binding, allowing proper positioning within the cell or conferring substrate specificity. The SH2 and SH3 domains mediate protein interactions and help to regulate enzymatic activity by stabilizing specific protein conformations. Expression of ALT-PTK6 led to a reduction of full length PTK6 YF protein tyrosine phosphorylation, as well as reduced phosphorylation of putative PTK6 substrates (Figure 2B). PTK6 activity is modulated by intramolecular interactions, including binding of its SH3 domain to the proline-rich SH2-catalytic domain linker region [47]. We were not able to detect stable association of ALT-PTK6 with full-length PTK6 protein (Figure 2B).

PTK6 is phosphorylated at several tyrosine residues including tyrosine 342, which enhances its catalytic activity [4], [47]. Phosphorylation of the carboxy-terminal tyrosine 447 negatively regulates PTK6 activity [47], [50], but since this residue is mutated to phenylalanine in the PTK6 YF mutant, decreased PTK6 tyrosine phosphorylation likely represents decreased activating phosphorylation. ALT-PTK6 also decreased association of PTK6 with tyrosine-phosphorylated proteins (Figure 2B and C). ALT-PTK6 might compete for substrate interactions mediated by the PTK6 SH3 domain, thus preventing PTK6 kinase from binding with interacting proteins and phosphorylating its substrates. ALT-PTK6 may also prevent other kinases from phosphorylating PTK6 if these associate through the PTK6 SH3 domain. We found that ALT-PTK6 associates with endogenous Sam68 and β-catenin in PC3 cells (Figure 2D), supporting the idea that ALT-PTK6 may affect signaling outcomes involving these proteins.

Activation of the Wnt pathway and β-catenin/TCF transcription contributes to cancer growth. Previously, we showed that β-catenin transcriptional activity is repressed by nuclear-targeted PTK6 [23]. Recently we showed that cytoplasmic PTK6 promotes growth of prostate cancer cells. Knockdown of endogenous PTK6 protein that is largely localized to the cytoplasm resulted in decreased PC3 cell proliferation. In contrast, reintroduction of PTK6 protein into the nucleus led to growth inhibition [25]. Here we show that ectopic ALT-PTK6 expression promotes nuclear translocation of the full length PTK6 protein (Figure 3C) and enhances the ability of PTK6 to repress transcription of a β-catenin/TCF reporter construct (Figure 3B). Accordingly, introduction of increasing levels of ALT-PTK6 led to decreased expression of endogenous β-catenin/TCF targets in PC3 cells (Figure 4). In addition, induction of ALT-PTK6 in PC3 cells led to reduced growth and colony formation (Figure 5). This is significant, because these data indicate that ALT-PTK6 has the potential to inhibit Wnt/β-catenin/TCF signaling and aberrant growth in prostate cancer.

**Figure 6:**
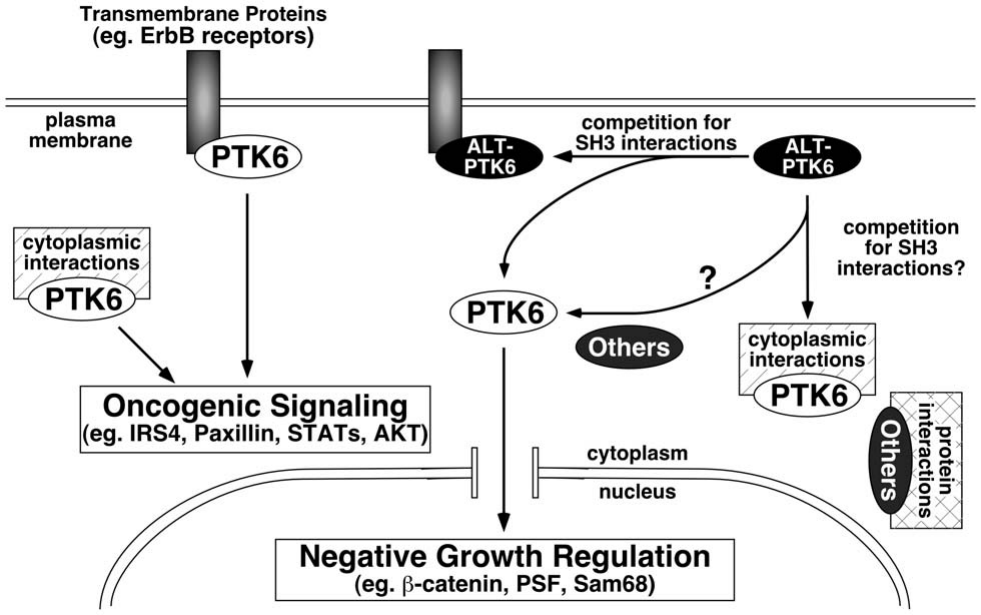
ALT-PTK6 may regulate activities of full-length PTK6. PTK6 may promote oncogenic signaling by phosphorylation and association with transmembrane and cytosolic proteins. ALT-PTK6 can compete for SH3 interaction with PTK6 and probably other proteins through its SH3 domain (and potentially the proline-rich carboxy terminal tail). In the case of PTK6 this results in reduced PTK6 tyrosine phosphorylation, reduced interaction with other tyrosine-phosphorylated proteins, reduced membrane association, and increased nuclear function; at least some of these factors contribute to growth regulation such as negative regulation of β-catenin/TCF signaling.

A possible model of how ALT-PTK6 might influence signaling is proposed in Figure 6. In cancer cells, PTK6 promotes oncogenic signaling at the membrane and in the cytoplasm through interactions with growth factor receptors and cytoplasmic substrates such as IRS4, paxillin, STATs, (reviewed in [1], [22]), and AKT [40]. Recently we found that PTK6 is able to directly phosphorylate and promote activation of cytoplasmic AKT [40]. When ALT-PTK6 is present (or if the balance shifts in the favor of ALT-PTK6) it can compete for SH3-mediated interactions of PTK6 substrates and binding partners. This competition may result in a reduction of membrane associated PTK6, and allow free pools of PTK6 to enter the nucleus, where it could mediate growth inhibitory functions by regulating β-catenin, Sam68, or PSF. ALT-PTK6 might also block associations of other proteins beside PTK6 through its SH3 domain, and perhaps also through the unique proline-rich carboxyl-terminus which is conceptually capable of interacting with unknown SH3 domains. The total effect of ALT-PTK6 expression in cancer cells may be negative growth regulation (Figure 5B and C). The SRC-related FYN tyrosine kinase was recently reported to have alternatively spliced isoforms that affect the SH2-kinase domain linker region that is recognized by the FYN SH3 domain, resulting in altered autoinhibition as well as altered SH3-mediated substrate recognition [59].

Our findings suggest that it is important to consider ALT-PTK6 when targeting PTK6 therapeutically. The ability of ALT-PTK6 to influence the intracellular localization of the full length protein and its ability to associate with and phosphorylate targets can influence cell signaling pathways and growth. Fully understanding the functions of ALT-PTK6 could aid in developing a small peptide that could specifically promote PTK6 nuclear functions, and restore regulation of nuclear PTK6 substrates such as Sam68 and PSF, as well as inhibiting β-catenin/TCF transcriptional activity in prostate cancer cells.

## Materials and Methods

### Ethics statement

The tissues from which primary cell cultures were derived were obtained with written informed consent under approval by the Institutional Review Board at Stanford University (protocol ID 13895) and in compliance with the Helsinki Declaration.

### Cell lines and tissue culture

Most cell lines including the human prostate adenocarcinoma cell lines PC3 and DU145, the human colon adenocarcinoma cell lines HCT116 and SW480, the embryonic kidney cell line HEK293, and the human hepatoma cell line HepG2 were obtained from ATCC (Manassas VA), and cultured as recommended by ATCC. The benign prostatic hyperplasia epithelial cell line BPH-1 (a gift from Dr. Simon Hayward (Vanderbilt University, Nashville TN)) was cultured in RPMI-1640 containing 5% fetal bovine serum, 100 U/ml penicillin and 100 µg/ml streptomycin [60]. Primary cultures from the peripheral zone of normal human prostate and from primary prostate adenocarcinomas were established and characterized according to previously described methods [61], [62]. Tetracycline-inducible PC3 cells were cultured with tetracycline-free fetal bovine serum (Gemini Bio-Products, West Sacramento CA).

For colony formation assays stable tetracycline inducible cells were serum starved for 48 hours and induced with 1 µg/ml tetracycline (Sigma, St. Louis MO) if applicable. Cells were then trypsinized, counted and 1.7×10^4^ cells seeded per well in a 6-well tissue culture plate and cultured as parental PC3 cells with tetracycline if appropriate. After 10 days the cells were fixed with ice cold methanol for 10 minutes and colonies were stained using 0.5% crystal violet.

### Plasmid constructs and stable cell lines

For ectopic expression of PTK6, the constitutively active pcDNA3-Myc-PTK6 YF (PTK6 YF) construct was used, which has the negatively regulatory tyrosine 447 mutated to phenylalanine. The pcDNA3 vector was used as a control and to keep total amount of plasmid DNA constant for co-expression experiments. Tetracycline inducible plasmid constructs were made using the pcDNA4/TO vector (Invitrogen, Carlsbad CA) linearized using HindIII endonuclease (Invitrogen). The 2.1 kb products of the HindIII digests of pcDNA3-Myc-ALT-PTK6 were gel extracted and ligated with the pcDNA4/TO vector backbone. Constructs were sequenced using CMV-forward (5′-CGCAAATGGG CGGTAGGCGT G-3′) and BGH-reverse (5′-TAGAAGGCAC AGTCGAGG-3′) primers. For generation of the GST-ALT-PTK6 expression construct, pGEX-2TK vector (GE Healthcare, Piscataway NJ) was linearized using Sma I and the gel extracted ALT-PTK6 fragment, which was excised from the expression construct using Xma I and Hind III followed by filling in overhangs, was ligated to the vector backbone in-frame with GST. GST-ALT-PTK6 protein was produced by transforming BL21 bacteria that were allowed to grow at 37°C to an optical density of 0.8-1.0 before induction with IPTG for 2 hours and purification using Glutathione sepharose 4B beads (GE Healthcare).

The PC3 human prostate tumor cell line was transfected with pcDNA6/TR (Invitrogen) using lipofectamine as per manufacturer's instructions. Cells were plated sparsely and transfected cells were selected with 2 µg/ml blasticidin (Invitrogen) over a two-week period. Single colonies of the PC3-TR (Tet Repressor) cells were isolated, expanded, and screened by transient transfection using pcDNA4/TO-Myc-ALT-PTK6 in the presence and absence of 1 µg/ml tetracycline (data not shown). A clone with no detectable expression in the absence of tetracycline was used for subsequent stable cell lines.

For stable tetracycline-inducible cells, PC3-TR cells were transfected with pcDNA4/TO-Myc-ALT-PTK6 and selected as above, adding 200 µg/ml of Zeocin (Invitrogen). Several clones were expanded for each cell line and screened with and without tetracycline addition by both immunoblotting and immunofluorescence staining to determine expression.

### Sequencing and PCR analysis of PTK6 RNA

RNA was isolated with TRIzol (Invitrogen) as per manufacturer's instructions. For generating cDNA, 5 µg of total RNA was reverse transcribed using random hexamer primers as per manufacturer's suggestion (Invitrogen). Two µL of the reaction was used to amplify PTK6 cDNA for 35 cycles at 72°C using the primers 5′-CCTGGGCCCC AAGTATGT-3′ (forward) and 5′-CAGAATTCCA TGGATGAAAG AGACACC-3′ (reverse). The products from the PCR were cloned into pBluescriptKSII(-), four colonies of transformed DH5α were picked, plasmids isolated, and sequenced. Sequences were aligned with PTK6 mRNA (Accession NM_005975).

Semi-quantitative PCR of cDNA samples of PTK6 transcripts was done using two sets of human PTK6 primers. Set 1: the forward primer 5′-GCTATGTGCC CCACAACTAC C-3′ specific to exon 1, and the reverse primer 5′-CCTGCAGAGC GTGAACTCC-3′ specific to exon 3; Set 2: the forward primer 5′-TGTGCCCCAC AACTACCTGG-3′ specific to exon 1, and the reverse primer 5′-TGCAGAGCGT GAACTCCTCC-3′ specific to exon 3. The PCR reaction was carried out using an annealing temperature of 63°C for 30 cycles. Human β-actin forward primer: 5′-AAAATCTGGC ACCACACCTT CTAC-3′, and reverse primer: 5′- TAGCACAGCC TGGATAGCAA CG -3′ were used to measure β-actin levels in cDNA samples as a control. PCR reactions were separated on 1.5% agarose gels and stained with ethidium bromide. Quantitation was performed using the public domain NIH ImageJ program [63].

Quantitative Real Time PCR amplification was done in triplicate using primers specific for PTK6 exon 2 (PTK6-X2_For 5′-CGGAACCGTG GTTCTTTG-3′ and PTK6-X2_Rev 5′-ACTCGGCTTC TCGCTGAC-3′) and exon 8 (PTK6-X8_For 5′-TGTTCCTGCT CTTCCCAGTT-3′ and PTK6-X8_Rev 5′-TGGGAGGAAA GAACCCTTGA-3′) as described previously [64]. The levels of PTK6 transcripts were normalized against the levels of cyclophilin mRNA which was used as an internal control, and for comparison of different exons, starting quantities were also normalized to a PTK6 plasmid control to adjust for variations in primer efficiencies between different exons.

### Immunofluorescent staining

Ten thousand PC3 cells were added per well on a 4-chamber culture slide (BD Biosciences, Bedford MA) and cells were allowed to attach for 24–48 hours. Cells were fixed with Carnoy's fixative (60% ethanol, 30% chloroform, 10% glacial acetic acid). Primary Myc-epitope antibody (Cell Signaling Technologies Inc., Boston MA) was diluted 1∶250 in 3% BSA (bovine serum albumin) in TNT buffer (100 mM Tris-HCl pH 7.5, 150 mM NaCl, 0.05% Tween 20), incubated at 4°C overnight, and then detected with fluorescein isothiocyanate (FITC)-conjugated anti-mouse IgG (Sigma). Cells were incubated for 5 minutes in 2 µg/ml 4′,6-diamidino-2-phenylindole (DAPI) (Sigma), rinsed twice with TNT buffer, and mounted with mounting media (Vector Laboratories Inc., Burlington CA).

### Fractionation, immunoblotting, GST pull-down assays and immunoprecipitation

Total cell lysates were harvested and immunoblotting was done as described previously [14]. PC3 total cell lysates were used for GST-pull down assays with GST and GST-ALT-PTK6 fusion proteins [14]. Fractionation of stable inducible ALT-PTK6 PC3 cells was carried out using the ProteoExtract Subcellular Proteosome Extraction Kit (Calbiochem, San Diego CA) as per manufacturer's instructions. Ten percent of the final volume for each fraction was used. Antibodies against Cyclin D1, E-cadherin, Neu/ErbB2, c-Myc, and Sp1 were obtained from Santa Cruz Biotechnology (Santa Cruz CA); anti-Myc-tag antibody was purchased from Cell Signaling Technologies; antibodies specific to β-actin and α-tubulin were obtained from Sigma; β-catenin specific antibody was purchased from BD Transduction Laboratories. For detection of phosphotyrosine proteins, a combination of anti-phosphotyrosine antibodies from Upstate/Millipore (4G10) and Santa Cruz (PY20) was used. For immunoprecipitation, 500 µg of total cell lysates were used as described previously [23], using 1 µg of mouse anti-Myc-tag (Cell Signaling Technologies Inc.) for pull-down. Quantitation of blots was performed using the public domain NIH ImageJ program [63].

### Luciferase reporter assay

Luciferase assays were conducted as described previously [23]. In brief, to determine the effects of ALT-PTK6 on β-catenin/TCF transcription, and transcriptional inhibition by PTK6, HEK293 cells were co-transfected with Super 8X TOPflash [51] (a gift from Dr. Randall Moon, University of Washington) containing eight TCF/Lef binding sites (TOPflash), β-catenin, PTK6 YF and ALT-PTK6. Renilla was used as a control for transfection efficiency to normalize values; vector was used to keep total amount of plasmid DNA constant. The transcriptionally dead Super 8X FOPflash (FOPflash) was used as a negative control. Luciferase activity was assessed 24 hours after transfection using the Dual-Luciferase Reporter Assay System (Promega, Madison WI) and the Clarity Microplate Luminometer (BioTek Instruments Inc., Winooski VT). At least three independent experiments were performed in duplicate.

## Supporting Information

Figure S1 Quantitative PCR of PTK6 transcripts containing exon 2 and exon 8. A) Quantitative real-time PCR analysis of PTK6 transcripts using primers specific to exon 2 (X2) of the full-length PTK6 transcript and exon 8 (X8) of ALT-PTK6 and PTK6 transcripts. B) Values for both exons were normalized to cyclophilin loading control and PTK6 expression construct (which contains exon 2 and exon 8 sequences in a 1∶1 ratio). C) The ratios of normalized PTK6 exon 8 to exon 2 were determined for each cell line, and do not show a particular trend with regards to the primary tumor site from which the cell lines were derived.
(0.10 MB TIF)
