# Supplementary figures and images for: The Alternative Splice Variant of Protein Tyrosine Kinase 6 Negatively Regulates Growth and Enhances PTK6-Mediated Inhibition of β-Catenin
Source: PLoS One. 2011 Mar 30;6(3):e14789. doi: 10.1371/journal.pone.0014789 (PMC3068133; doi:10.1371/journal.pone.0014789)

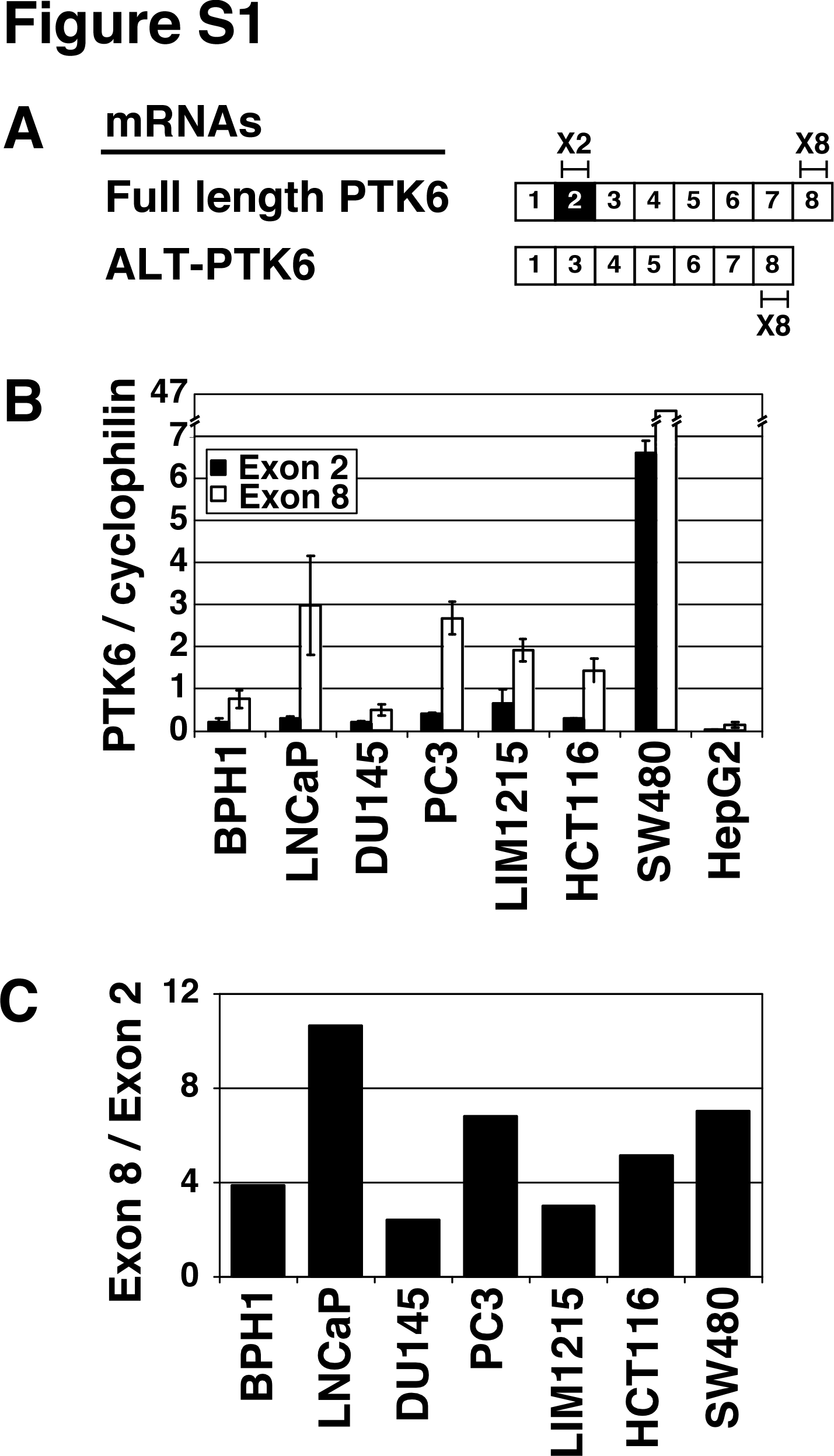

Supplement: Figure S1 — Quantitative PCR of PTK6 transcripts containing exon 2 and exon 8. A) Quantitative real-time PCR analysis of PTK6 transcripts using primers specific to exon 2 (X2) of the full-length PTK6 transcript and exon 8 (X8) of ALT-PTK6 and PTK6 transcripts. B) Values for both exons were normalized to cyclophilin loading control and PTK6 expression construct (which contains exon 2 and exon 8 sequences in a 1∶1 ratio). C) The ratios of normalized PTK6 exon 8 to exon 2 were determined for each cell line, and do not show a particular trend with regards to the primary tumor site from which the cell lines were derived. (0.10 MB TIF) [file pone.0014789.s001.tif]
